# Native mass spectrometry meets X-rays for the elucidation of protein structures

**DOI:** 10.1042/BST20250111

**Published:** 2026-05-08

**Authors:** Thomas Kierspel, Emiliano de Santis, Kira Schamoni-Kast, Clement Blanchet, Erik G. Marklund, Carl Caleman, Charlotte Uetrecht

**Affiliations:** 1 CSSB Centre for Structural Systems Biology, Deutsches E­lektronen-Synchrotron DESY & Leibniz Institute of Virology (LIV) & University of Luebeck, Notkestraße 85, 22607 Hamburg, Germany; 2 Department of Physics and Astronomy, Uppsala University, Box 516, 75120 Uppsala, Sweden; 3 Department of Chemistry for Life Sciences, Uppsala University, 75123 Uppsala, Sweden; 4 Department of Physics, University of Rome Tor Vergata and INFN, via della ricerca scientifica 1, Rome 00133, Italy; 5 University of Luebeck, Institute of Chemistry and Metabolomics, Ratzeburger Allee 160, 23562 Lübeck, Germany; 6 European Molecular Biology Laboratory (EMBL), Hamburg Unit c/o DESY, Notkestraße 85, 22607 Hamburg, Germany; 7 Center for Free-Electron Laser Science, Deutsches Elektronen-Synchrotron DESY, Notkestraße 85, 22607 Hamburg, Germany

**Keywords:** Native mass spectrometry, simulations, small-angle x-ray scattering, viral proteins

## Abstract

Small-angle X-ray scattering (SAXS) is a powerful and widely used technique for structural biology, providing information about solution structures, i.e., without the need for crystallization or cryogenic conditions. However, its applicability is limited in cases where sample heterogeneity, conformational mixtures, or aggregation are present. While modern BioSAXS routinely employs inline purification (e.g., SEC-SAXS) to address heterogeneity, gas-phase SAXS, enabled by native mass spectrometry as a sample delivery method, offers an even higher degree of population selectivity by providing well-defined and mass-selected ion populations. The principal challenge of this approach is the inherently low particle density in the gas phase. In the present overview, we present simulations of gas-phase SAXS viral nonstructural proteins and capsids and compare them against diffraction patterns from prototypical GroEL. We evaluate the expected scattering signal under experimentally realistic conditions and discuss potential implementations and use cases at both synchrotron radiation sources and X-ray free-electron lasers, thereby outlining regimes in which gas-phase SAXS may become a viable complementary tool for structural studies.

## Introduction

Structure and function of biomolecules are tightly connected, driving extensive efforts to determine protein structures and extract mechanistic insights. Small-angle X-ray scattering (SAXS) provides structural information on biomolecules in solution under near-native conditions [1], complementing high-resolution methods such as crystallography and more recent methods such as cryogenic electron microscopy (cryo-EM) [2]. The use of SAXS in biology has expanded significantly, supported by advances in synchrotron automation and beamline performance [3,4]. The present work outlines how SAXS can be extended to highly heterogeneous systems by performing scattering on biomolecules in the gas phase. Such capability is especially valuable when considering proteoforms, whose numbers may reach millions in humans [5]. Their coexistence, due to equilibria, gene splicing, or variable modification patterns, leads to heterogeneity that complicates crystallography, cryo-EM, and conventional solution SAXS, which is particularly sensitive to size mixtures [6–8], since the recorded SAXS signal averages over all species, thereby limiting interpretability.

Gas-phase SAXS offers distinct advantages by enabling size- and conformation-selective sample delivery through integration with native mass spectrometry (nMS) [9], while largely preserving solution-like biomolecular structures. This ability of nMS has been demonstrated, for example, via ion mobility mass spectrometry (MS) [10] or cryo-EM experiments subsequent to nMS [11,12] for soluble proteins and also membrane proteins [13].

One particular advantage of gas-phase SAXS is the absence of solvent surrounding the sample, which improves contrast due to reduced background and substantially mitigates radiation damage originating from radical-induced aggregation in solution. In the gas phase, radiation damage requires multiple coincident ionization events, which can be neglected at synchrotron sources based on X-ray absorption cross-section estimates or as seen in nMS-X-ray spectroscopy experiments [14].

The main limitation for gas-phase SAXS of biomolecules is the low particle density in the interaction region with the X-rays. This can partially be mitigated by technologies to improve the ion beam density (see below) or by utilizing the high brightness of modern X-ray sources, namely X-ray free-electron lasers (XFELs).

XFELs have already been used for SAXS measurements on ultra-dilute (<0.1 mg/ml) samples in solution [15] and are currently being explored for gas-phase single-particle imaging (SPI) approaches [16–18]. In contrast with SPI, gas-phase SAXS does not require a tightly focused X-ray beam and the selection of millions of high-quality hits, since each pulse probes a large ensemble of biomolecules and contributes equally to the signal. While the resulting diffraction patterns contain less structural information than those obtained by SPI, gas-phase SAXS is experimentally less demanding, owing to its relaxed requirements on both the ion and X-ray beams. The total scattering signal in SAXS is determined by the number density of the biomolecules in the gas phase, the interaction volume, and the total number of incident photons. Under typical conditions, where the ion beam exceeds the X-ray beam in cross-section, a larger X-ray focus reduces the scattering per biomolecule, which is compensated by the larger number of illuminated particles. This can provide advantages by reducing radiation damage to the biomolecules, which is especially important for smaller (<100 kDa) protein complexes [14] that remain in addition too small for X-ray-based SPI experiments [18].

Experimentally, the required ion beam parameters are accessible using sample delivery systems such as the *MS SPIDOC* prototype [19,20]. Originally developed for SPI experiments, the system employs nano-electrospray ionization (nESI) to transfer structurally intact biomolecular complexes into the gas phase. It incorporates an aerodynamic lens-based ion-transfer interface that increases the ion beam density of large biomolecules by more than an order of magnitude [21,22]. Further, it integrates MS components, including a digitally controlled ion filter and ion trap [23,24], as well as a drift cell for ion mobility [25], all specifically optimized for large biomolecular complexes. Finally, the transport to the interaction region occurs under high vacuum conditions via electric fields, eliminating the need for seeding or focusing gases.

## Current challenges

The main challenge for gas-phase diffraction experiments such as SAXS results from the very low sample density associated with ion beams. In this regime, gas-phase sample delivery systems, which require seeding gas such as supersonic gas jets [26,27] or aerodynamic lenses [28] are often limited by the carrier gas itself. Furthermore, the residual gas present in high-vacuum environments can make a significant contribution and become the dominant background. This is especially true for SAXS, where at small scattering angles the scattering from an individual particle scales approximately with the square of its number of electrons, whereas the total scattered intensity from an ensemble of particles scales linearly with the number of such particles. Thus, residual gas molecules present along the entire X-ray beam path can generate a substantial total number of detected background photons. In contrast, biomolecular ions exhibit a much larger per-particle scattering cross-section but interact with the X-ray beam only within a small interaction region. The achievable signal-to-background ratio is hence strongly influenced by the overall vacuum level, X-ray beam collimation, suppression of parasitic X-ray scattering upstream of the interaction region, and efficient stopping of the direct beam close to the interaction point. These factors directly impact the required exposure times and beam intensities needed to reach a detectable signal. Yet, the feasibility of gas-phase SAXS at synchrotron sources has already been demonstrated under slightly different conditions, namely non-natively sprayed biomolecules stored in an ion trap [29].

## Simulated SAXS diffraction pattern and discussion

**Figure 1:**
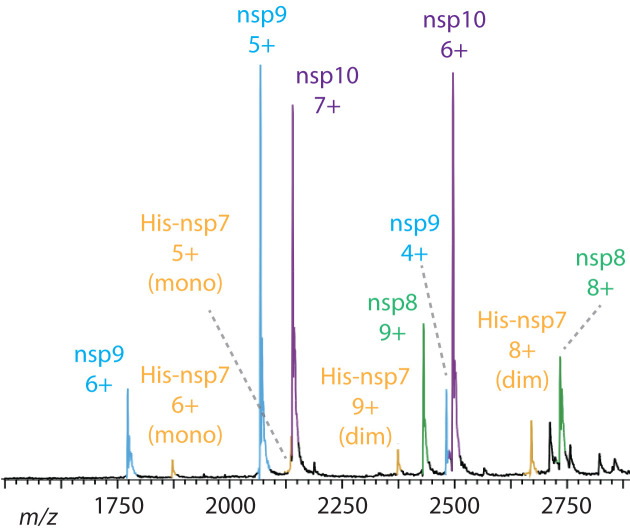
Representative Mass Spectrum in nMS Heterogeneous mass spectrum of the processed SARS-CoV partial polyprotein His-7–10, consisting of nonstructural proteins (nsp), adapted and modified from [30] (licensed under CC BY 4.0). Different charge states are indicated by numbers, and the various species including monomers (mono) and dimers (dim) are highlighted in different colors.

Figure 1 shows a heterogeneous mass spectrum of the processed SARS-CoV partial polyprotein, consisting of the monomeric and dimeric nonstructural proteins (nsp), adapted and modified from [30] (licensed under CC BY 4.0). Different charge states are indicated by numbers, and the various species are highlighted in different colors. Such heterogeneous mass spectra are common in biological systems and often form the basis of nMS experiments, as the complex interplay between different species provides valuable insight into underlying biological processes [9,31]. Using MS techniques—such as quadrupole mass filters, ion mobility, or charge-state reduction (e.g., via detergents) [32–34]—it is possible to tune charge-state distributions, isolate individual or multiple adjacent peaks in the mass spectrum, and further separate ions based on their conformations. These purified samples can then serve as ideal starting points for SAXS experiments.

**Figure 2:**
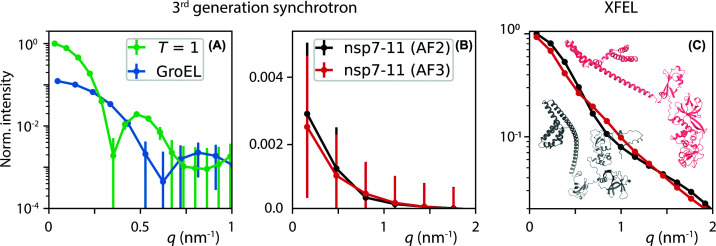
Simulated Gas-Phase SAXS Diffraction Patterns Simulated gas-phase SAXS diffraction patterns of (**A**) the *T* = 1 empty norovirus capsid, the chaperonin GroEL, and (**B, C**) two competing AlphaFold structure prediction models (AF2 and AF3) of the unprocessed SARS-CoV-2 partial polyprotein nsp7–11. The simulations are shown for two experimental conditions, a third-generation synchrotron (A,B) and an XFEL (C) and are normalized to the maximum scattering intensity of *T* = 1 for the synchrotron and nsp7–11 (AF2) for the XFEL. Ribbon models of nsp7–11 (AF2 and AF3) are overlaid in panel (C) for structural comparison.

Figure 2 shows simulated SAXS diffraction patterns of three representative size regimes of protein complexes, namely, the (unprocessed) SARS-CoV-2 partial polyprotein consisting of nsps 7–11 [35], the larger chaperonin GroEL, and *T* = 1 (triangulation number) empty norovirus capsids from the GII.2 Snow Mountain virus. The experimentally observed most abundant charge states, as determined by nMS, for GroEL, the nsp7–11, and different variants of the *T* = 1 norovirus capsids are summarized in Table 1 [14,35,36]. The structures of GroEL and *T* = 1 particles are treated as gas-phase structures but are based on structures previously determined by X-ray crystallography and/or cryo-EM [37,38]. The nsps are connected through flexible linkers, hampering high-resolution techniques. Structural models of nsp7–11 have so far been obtained using AlphaFold 2 (AF2) [39] and AlphaFold 3 (AF3) [40], resulting in competing models.

**Table 1:** Protein systems and simulation parameters for SAXS diffraction

	nsp7–11	GroEL PDB ID: 1ss8	*T* = 1 PDB ID: 6ouc
Mass	60 kDa	808 kDa	3.3 MDa
*m/z*	4300	12 600	22 760
	*z* = 14	*z* = 64	*z* = 145
Density [ion/cm^3^]	1 × 10^6^ (1 × 10^7^)	2.2 × 10^5^ (2.2 × 10^6^)	9.7 × 10^4^ (9.7 × 10^5^)
Counted photons/mJ	0.0025 (0.025)	0.06 (0.6)	0.4 (4)

Molecular weight, charge state (*z*), ion beam density, and diffracted photon counts are listed, which were used to simulate the SAXS diffraction pattern in Figure 2. Numbers in parentheses correspond to recent instrumental developments that increase the ion beam densities [21,22].

The SAXS diffraction patterns were calculated using the CONDOR simulation package [41] (see SI) with representative beamline parameters, including a photon energy of 10 keV and a large-area photon counting detector placed 4.5 m from the sample, yielding access to scattering vectors up to *q* = 3.8 nm^−1^. Error bars represent the standard error of the mean (SEM) based on a Poisson error model implemented by the pyFAI [42] Python package. Within this framework, the uncertainty depends solely on the estimated ion beam density and the incident X-ray pulse energy.

To assess experimental feasibility within the limited time available at a large-scale facility, simulated intensities were scaled to 10 (1) h acquisition time at a synchrotron source, with 10 h representing an achievable goal for beamtime. In contrast, 100 (10) min was assumed for European XFEL simulations to illustrate the performance during a comparatively short measurement time at a free-electron laser. Values in parentheses correspond to recent instrumental developments that significantly increase ion beam densities in nMS by improving solution-to-gas-phase transmission efficiency [21,22] as implemented in *MS SPIDOC*. Further simulation parameters, including details of the estimated ion beam densities, are summarized in Table 1.

Considering the synchrotron case first, the simulations indicate that gas-phase SAXS at synchrotron sources can resolve the overall size and shape of biomolecules, providing access, for example, to their global domain organization. In contrast, distinguishing more subtle structural differences—such as those between the two AlphaFold models considered here—does not appear feasible within the estimated experimental conditions.

However, synchrotron-based gas-phase SAXS provides substantially more structural information than techniques such as ion mobility MS, since the latter reports only a single orientationally averaged collision cross section. By capturing the full scattering profile, SAXS diffraction patterns enable finer discrimination between alternative conformations or assembly states.

In general, the simulations are consistent with the expected higher scattering intensity at low *q* for larger biomolecular complexes. They also indicate an increase in the SEM for smaller biomolecules, which should be interpreted with caution. The relative density scaling applied here assumes a constant ion current, whereas in native MS, molecular mass scales with volume and charge state with surface area. Thus, under this assumption, larger biomolecules inherently generate a stronger scattering signal, leading to a correspondingly lower SEM. This does not necessarily reflect the real relative ion beam density of biomolecules, which is difficult to estimate *a priori* due to its strong dependence on experimental conditions such as concentration, ionization efficiency, or *m/z*-dependent instrumental transfer functions.

Notably, the simulations for nsp7–11 at the synchrotron underestimate the achievable performance of gas-phase SAXS in an ion trap; see the experimental results reported in [29]. Here, the even smaller cytochrome *c* ions (≈12 kDa), electrosprayed under non-native conditions and probed within an ion trap, yielded measurable scattering signals.

Gas-phase SAXS experiments at XFELs, such as the European XFEL, can extend this technique into a highly powerful tool for structural biology, as they enable the detection of subtle structural differences. This capability is exemplified by the two distinct AlphaFold models (AF2 and AF3) of nsp7–11, which yield statistically distinguishable scattering profiles with error bars smaller than the symbol size. When combined with simulations, these measurements would allow structural insights into tertiary-structure arrangements even at relatively low scattering resolution.

The simulations in Figure 2 represent an idealized scenario, in which background contributions are neglected and fixed biomolecular structures are assumed. In practice, biomolecules—especially polyproteins—are flexible and may adopt multiple conformations, such that the measured scattering would represent an ensemble average rather than a single structure. Scattering from residual gas and parasitic beamline effects will also reduce the achievable signal-to-background ratio, particularly at low *q*. Instead of applying a generic background model, Table 1 reports the number of diffracted photons per mJ of incident pulse energy for the given ion beam density. These values provide a basis for beamline-specific estimates of the tolerable background at each scattering angle while still allowing a measurable signal.

## Conclusion and potential science cases

From our simulations and the reported experiment [29], we infer that developing MS-based gas-phase SAXS for diffraction experiments has the potential to become a useful tool for structural biology. Synchrotrons are considered as the working horse, where the overall size and conformation of a biomolecule could be determined. XFELs, on the other hand, could provide very targeted information, for example, on binding sites, especially in combination with AI-based structure prediction models such as AlphaFold. To more specifically illustrate the potential of gas-phase SAXS at synchrotrons or XFELs, we highlight four specific types of systems in addition to the simulations where this development would be particularly impactful.

### Virus assembly intermediates in the example of noroviruses

Noroviruses assemble into icosahedral capsids, where the triangulation number *T* defines the number of subunits and quasi-equivalent positions. Native particles are *T* = 3 with 180 VP1 (major capsid protein) copies [43,44], but *T* = 1 particles can form under alkaline conditions or after *N*-terminal truncation [43,45]. Native ion mobility MS has probed assembly intermediates, whose collision cross sections best matched a partial five-fold vertex, suggesting a plausible nucleation route [44]. This implies that both *T* = 1 and *T* = 3 particles may emerge from similar nucleation events, with conditions steering the final size distribution. Ion mobility resolution and the available structural models are limited, however. Solution SAXS, on the other hand, requires a higher (>1 mg/ml) protein concentration compared with nESI (<1 mg/ml), which can lead to kinetically trapped states. It remains, hence, unclear if intermediates reported from SAXS for bovine norovirus are on pathway [46]. Gas-phase SAXS combined with structure-prediction tools like AlphaFold would distinguish candidate intermediates more clearly and improve understanding of whether capsid size is fixed at nucleation or determined later.

### Amyloids

Amyloids form diverse oligomeric intermediates on route to fibrils, and small oligomers are implicated as cytotoxic species in neuropathology [47]. While fibrils are accessible by cryo-EM, early oligomers are not and are mainly studied by MS [48]. Integrating MS with gas-phase SAXS would yield far better structural insight into these transient intermediates and the mechanisms of fibril formation with distinct structures [49].

### Viral glycoproteins

Enveloped viruses carry membrane-embedded glycoproteins essential for attachment, entry, and fusion [50,51]. Extensive glycosylation shields these proteins from immune recognition, but the flexibility of the glycans hampers structural analysis in cryo-EM and X-ray crystallography. Conventional SAXS can probe glycan ensembles but is limited by the membrane environment. Small angle neutron scattering with stealth discs can address this [52], though it is complex and costly. nMS can remove membrane components, enabling structural characterization of released proteins [53], even directly from, e.g., mitochondrial membranes [54]. Gas-phase SAXS would extend these capabilities by providing global structural information without membrane interference.

### Human plasma glycoproteins such as serotransferrin

Human serotransferrin transports Fe^3+^, contains 19 disulfide bonds and up to three *N*-linked glycans, and serves as an acute inflammation biomarker [55]. MS-based analysis enables the characterization of disease-associated glycoforms of serotransferrin [56]. Hence, this would be an interesting test case for gas-phase SAXS on minimally purified samples, allowing assessment of whether post-translational modifications (PTMs) or single nucleotide polymorphisms cause conformational changes that could alter phenotypes. It is currently unclear how the glycan structures are affected by the gas-phase conditions. Soft-landing experiments with electrosprayed glycans and subsequent scanning tunneling microscopy show that even mild activation can bring about structural rearrangements [57], suggesting that SAXS data for glycoproteins are sensitive to experimental conditions. SAXS probes structures more deeply than other gas-phase techniques such as ion mobility and is therefore a promising means for revealing how glycans respond to being transferred to the gas phase. Notwithstanding the volatility of glycan structures, electrosprayed proteins are thought to be kinetically trapped near their solution conformations [58], implying that even if glycans collapse, their impact on protein conformation can remain. Indeed, ion mobility MS has shown that glycan identity has a notable impact on the structure of the glycosylated DC-SIGN carbohydrate recognition domain [59]. This example implies that gas-phase SAXS could be an avenue toward understanding the interplay between PTMs and protein structures and their role in biology.

### SI: simulation parameter

SAXS diffraction patterns were simulated from atomic coordinates using the CONDOR simulation package [41]. Protein structures were provided as PDB files and included GroEL (PDB ID: 1ss8), a geometrical configuration of norovirus-like particle capsids *T* = 1 (PDB ID: 6ouc), and two models of SARS-CoV-2 nsp7–11 (AF2 and AF3). The atomic models for AF2 and AF3 were obtained from AlphaFold predictions. For each system, 500 individual diffraction patterns were computed assuming elastic X-ray scattering from randomly oriented particles in vacuum, and the resulting intensity distributions were averaged to obtain the final SAXS pattern. The X-ray photon energy was set to 10 keV, at a pulse energy of 1 mJ, a circular beam focus with a diameter of 200 μm, and an ion beam width of 1 mm. Diffraction was recorded on a detector geometry matching a Pilatus 6M configuration, with a pixel size of 172 μm and an active area of 2463 × 2527 pixels. The sample-detector distance was fixed at 4.5 m. The simulations have been scaled to match the measurement time and the expected X-ray power at the synchrotron (16 mJ/s, EMBL P12 PETRA III) and the European XFEL (2 mJ, 27 000 pulses/s, SPB/SFX instrument).

### SI: AlphaFold 2 and AlphaFold 3

The models used here reflect the polyprotein sequence of nsp7–11 of SARS-CoV-2 (NCBI LOCUS NC_045512, isolate Wuhan-Hu-1). The polyprotein sequence of nsp7–11 was submitted to AlphaFold 2 standard run (20 cycles) [39] and AlphaFold3 (AF3) [40] standard settings. For AF2, the best model (model 4) was picked with a predicted template modeling score (pTM score) of 0.312. For AF3 the best model (model 0) was picked with a pTM score of 0.33. This score provides an overall estimate of the accuracy of the predicted protein structure, ranging from 0 to 1 [39,60].

## Perspectives

- Highlight the importance of the field: Proteins make up complex systems, with multiple coexisting proteoforms, stoichiometries, and other kinds of heterogeneity, which limits conventional techniques for structural biology. Using the non-destructive separation in nMS to deliver proteins for downstream diffraction with SAXS is an avenue toward structure elucidation of specific proteins from complex mixtures and a substantially more comprehensive view of the structural complexity of protein systems.
- Summary of the current thinking: The question remains if gas-phase SAXS from dilute samples as subjected by nMS is feasible. Here, calculations are presented suggesting that such experiments can indeed be conducted at highly brilliant X-ray sources such as XFELs as well as third-generation synchrotrons.
- Comment on future directions**:** Combining nMS with SAXS has the potential to advance our knowledge of many protein systems. Some examples where its impact will be most notable are (I) polydisperse systems, including self-assembling proteins, such as virus capsids and amyloids, where the multitude of assembly intermediates are challenging or impossible to capture with X-ray crystallography, cryo-EM, and other techniques; and (II) heavily modified proteins, such as glycoproteins, which play key roles in viral infections and many other systems associated with health and disease. To enable such investigations, efforts need to be put into the technical development, testing, and integration of nMS-enabled SAXS at synchrotron beam lines.

## Open Access

Open access for this article is provided via our Subscribe to Open programme, made possible through the support of our subscribing institutions. Learn more here: https://portlandpress.com/pages/open_access_options_and_prices#conditional.

## Abbreviations

AF2: AlphaFold 2
AF3: AlphaFold 3
cryo-EM: cryogenic electron microscopy
MS: mass spectrometry
nESI: nano-electrospray ionization
nMS: native mass spectrometry
nsp: nonstructural proteins
pTM score: predicted template modeling score
SAXS: small-angle X-ray scattering
SEM: standard error of the mean
SPI: single-particle imaging
XFELs: X-ray free-electron lasers
